# Investigating on the influence mechanism of sausage of sea bass on calcium absorption and transport based on Caco-2 cell monolayer model

**DOI:** 10.3389/fnut.2022.1046945

**Published:** 2022-10-18

**Authors:** Zhongqiang Wang, Ranzhuo Ma, Zhihui Jia, Peng Lin, Zhenhua Zhao, Wei Wang, Shumin Yi, Xuepeng Li, Jianrong Li

**Affiliations:** College of Food Science and Technology, National & Local Joint Engineering Research Center of Storage, Processing and Safety Control Technology for Fresh Agricultural and Aquatic Products, National R&D Branch Center of Surimi and Surimi Products Processing, National and Local United Engineering Lab of Marine Functional Food, College of Mathematical Sciences, College of International Education, Bohai University, Jinzhou, China

**Keywords:** sea bass sausages, proteomics, Caco-2 cell, calcium ion transport, pathway analysis

## Abstract

A monolayer Caco-2 cell model was established to explore the effects of sea bass sausage digestive juice containing phosphate on calcium ion transport. Differential proteins of Caco-2 cells treated with fish sausage juice were detected and analyzed by gene ontology (GO) functional annotation and kyoto encyclopedia of genes and genomes (KEGG) pathway analyses. Results revealed that after treatment with 0.23 mg/mL digestive juice of perch sausage *in vitro*, Caco-2 cell viability was the highest at 72 h (99.84%). Additionally, 0.23 mg/mL digestive juice of perch sausage *in vitro* significantly increased calcium ion transport. The transfer volume was 1.396 μg/well. Fish sausages containing phosphate significantly affected the protein expression levels of Caco-2 cells. Two hundred one differential proteins were detected, including 114 up-regulated and 87 down-regulated proteins. The main differential proteins included P02795, Q9P0W0, Q96PU5, Q9GZT9 and Q5EBL8. The adjustment ratios of the fish sausage group were 0.7485, 1.373, 1.2535, 0.6775, and 0.809, respectively. The pathway analysis showed that phosphate affected calcium ion absorption and transport through the P02795 enrichment pathway. The fish sausage group showed that the immune-related functions of cells were affected. This study expounds the effects of water-retaining agents on the nutritional quality of aquatic products and provides theoretical support for the research and application of surimi products.

## Introduction

Surimi is a concentrate of myogenic fibrous proteins obtained by completely removing fish bones, fins, and viscera, and continuously washing the fish flesh (1, 2). Good quality surimi and surimi products have good gel strength, sensory quality, and economic value. However, the current moisture content of commercially available surimi in China is 73–80% and is susceptible to juice loss during freezing, refrigeration, storage, and transportation (3). The addition of food additives is an effective method for improving the quality of the gel of surimi products, among which, phosphate complex is widely used as it improves the water-holding capacity of these products and prevents protein denaturation (4). Phosphate compounds have been widely used as additives in fish and seafood to improve the functional properties of these products by increasing the water retention capacity of fresh fish and reducing the thawing loss of frozen fish (5). When fish die at a low pH, the addition of phosphate increases the pH and thereby avoids the isoelectric point of proteins. The charges then repel each other, leaving more space between proteins, and moisture is retained in the muscle (6).

Calcium is the most abundant mineral found in the human body, which forms teeth and bones and participates in various physiological activities as a second messenger of cellular activities (7). Calcium homeostasis disorders induce the risk of bone diseases, metabolic diseases, and epithelial tumors (8, 9). Dietary calcium generally exists in bound form, which is decomposed into calcium ions under the action of gastric acid (10). Ionized calcium is found mainly in the duodenum and upper jejunum and is absorbed by the human body through active or passive transport and enters the blood through intestinal epithelial cells (11). Phosphorus and calcium homeostasis play important roles in many physiological systems and disorders in calcium and phosphorus metabolism lead to serious consequences, such as bone-related and cardiovascular diseases, which can be life-threatening (12).

Due to their spontaneous differentiation into small intestinal phenotypes and ability to maintain proton gradients (13), Caco-2 cells have been widely used in medicine and food as a cell model for the study of drug transport, metabolism, and toxicity (14). In a specific culture environment, Caco-2 cells carry out biochemical and morphological differentiation *in vitro* during the early stages of culturing, producing microvilli and enzymes related to the brush margin epithelium in the small intestines (15). The Caco-2 cell monolayer shows brush-like characteristics after close fusion, which forms a good carrier transport system. Differentiated Caco-2 cells have better morphological and functional differentiation than other colon cancer cell lines (16, 17). Therefore, the Caco-2 cell model can partly reveal the transport mechanism of nutrients or drug molecules in the human intestinal tract.

Proteomics refers to the characterization of the proteome (18), which is an important method for understanding gene functions (19). Changes in gene expression levels can be elucidated by analyzing the transcriptome or proteome (20). As the main regulator of life activities, the expression levels of proteins are closely related to the corresponding mRNA and host translation regulation, thus, proteomics is the most relevant method through which the biological system can be characterized (21). Tandem quality labeling (Tandem Mass Tags, TMT) is a proteomic quantitative method (22). By specifically labeling the amino groups of peptide ends and side chains, and after tandem mass spectrometry (MS/MS) analysis, the relative protein contents in 2, 6, or 10 groups of different samples can be simultaneously compared. It has the advantages of high throughput, high resolution, accurate protein quantification, good repeatability, and rich data (23); thus, it is suitable for a wide range of sample markers, including cells and tissues, and also has a fast reaction speed and high labeling rate. Moreover, TMT is widely used in disease marker screening, disease pathogenesis research, drug target research, and physiological and pathological research of animals and plants (24–31).

In this study, a monolayer Caco-2 cell model was established to explore calcium ion absorption and transport in surimi products. The differential proteins of Caco-2 cells treated with compound phosphate were detected by TMT protein quantitative technology using David software. The identified differential proteins were detected by GO functional annotation and KEGG pathway analyses. This paper preliminarily expounds the effects of water-retaining agents on the nutritional quality of aquatic products, as well as provides nutritional theoretical support for the research, development, and application of surimi products.

## Materials and methods

### Materials

Dulbecco's modified eagle medium (DMEM) (Gibco Co., USA); 0.25% trypsin-ethylene diamine tetraacetic acid (EDTA) solution (Gibco Co., USA); fetal bovine serum (Biological Industries Co., Israel); phosphate buffered saline (PBS) (Beijing Solebao Technology Co., Ltd., Beijing, China); Caco-2 cell line (Cell Resource Center, Institute of Basic Medicine, Chinese Academy of Medical Sciences, China); penicillin-streptomycin (Sigma, USA); Cell Counting Kit-8 (CCK-8) (Biyuntian Biology Co., Ltd.,). cell culture plate, cell culture bottle (Wuxi Ness Biotechnology Co., Ltd.,); DL-Dithiothreitol (DTT) (Chemical Pure Plomag Biotechnology Co., Ltd., Beijing, China); iodoacetamide (IAM) (Chemical Pure Promeg Beijing Biotechnology Co., Ltd., Beijing, China).

### Sample pretreatment

Fresh sea bass was used to make surimi by adding salt and 0.5% compound phosphate for vacuum chopping, then exhausting the enema. The simulated *in vitro* digestion of sea bass sausages was carried out by stimulating the digestive system of a bionic dynamic human stomach *in vitro*. An appropriate amount of digestive juice from sea bass sausages *in vitro* was obtained and freeze-dried for 72 h. Dry powder of the digestive juice was added to the DMEM cell culture medium according to the set concentrations; the complete cell culture medium was prepared. The control group consisted of a complete cell culture medium. Positive control group: consisted of cells and complete medium, in which complete medium contained 1 mg/L potassium phosphate, The experimental groups were based on a numerical range of normal human blood phosphorus levels and concentrations of freeze-dried powder of sea bass sausages *in vitro* (referred to as digestive juice of sea bass sausages simulated *in vitro*). Normal blood phosphorus levels in humans range from 0.74 to 1.39 mmol/L (32). The experimental groups were set to 0.11 mg/mL (0.06 mg/L), 0.15 mg/mL (0.09 mg/L) and 0.23 mg/mL (0.13 mg/L) (0.06 mg/L, 0.09 mg/L and 0.13 mg/L represent the concentrations of phosphate obtained from the freeze-dried powder of sea bass sausages).

### Culture of Caco-2 cells

After resuscitation, Caco-2 cells were transferred to a cell culture flask and supplemented with a culture medium. After gentle shaking and mixing, the cells were cultured at a constant temperature in a cell incubator; the culture medium was changed according to the cell growth state. Cell passage was carried out when the cell density reached 80–90%. The mouth of the culture bottle was disinfected with alcohol and transferred to a super-clean worktable. Then, the original medium was disposed and cells were washed with PBS two or three times to remove dead cells and cells in bad condition. Trypsin was added for digestion for 2–3 min. Subsequently, the cells were observed and a medium was added to stop digestion. When cells were digested and retracted, but not completely slipped off, cells that were still attached to the wall of the cell culture bottle were gently blown down and repeated as needed to make the cell suspension. The digested cells were sub-cultured in flasks in appropriate proportions and the complete culture medium was supplemented to the required amount. The Caco-2 cell culture flasks were placed in a cell culture box at 37°C and 5% CO_2_ for further experimentation (33).

### Effects of fish sausage digestive juice on Caco-2 cell activity

Cells with a density of 80–90% after inoculation were digested, which were then inoculated on a culture plate by adjusting the cell density. All experimental groups were set up at the same time. After cells adhered to the wall, the cell culture plates were removed and the original medium was discarded. Complete cell culture mediums in the same volume were added to the blank and control groups. The cell culture medium containing the highest phosphate concentration was added to the positive control group. In the experimental groups, the complete culture medium containing digestive juice with different phosphate concentrations was added. Five multiple holes were set up for each group and placed in the incubator to continue culturing. After incubating for a fixed time, the culture plates were removed and a corresponding volume of CCK-8 determination reagent was added according to the volume of the cell culture medium of each well. Then, plates were shaken evenly by the cross method and placed in a CO_2_ cell incubator under complete darkness. The optical density (OD) was measured at a wavelength of 450 nm by an enzyme labeling instrument. The cell activity was calculated as follows:


$$Cell\;activity=\frac{As-Ab}{Ac-Ab}\times 100\%$$

where As is the experimental well, containing cell culture medium, CCK-8 and digestion solution; Ac is the control well, containing the medium of the cells and CCK-8, but without the digestion solution; Ab is a blank well, which is medium without cells and digestive juices, but containing CCK-8.

### Establishment of Caco-2 cells absorption model

When the density of Caco-2 cells reached 80%, the original culture medium was discarded and the cells were gently rinsed with PBS two or three times to remove dead cells and cells in bad condition. Then, trypsin was added for digestion, and the digested cells were added to the top of a Transwell cell transport chamber, followed by adding a fresh cell culture medium to the bottom. The liquid was changed every other day during the initial stage of cell inoculation and every day after 1 week. The blank group contained the same amount of buffer. After cells were cultured for 21 d, cell differentiation was observed under an inverted microscope. During the culture period, the transmembrane resistance (TEER) values and transmittance of sodium fluorescein were measured regularly to determine whether the model was successful.

### Evaluation of Caco-2 cells absorption model

#### Morphological observation of Caco-2 cells

After culturing for 21 d, the liquid on both sides of the Transwell chamber was discarded and cells were washed with PBS. Pentanediol solution was added to the cells on both sides of the chamber to fix the cells. The polyester film was cut into a 0.5 × 0.8 cm rectangle, rinsed twice with 0.1 mol/L buffer (10 min each time), and fixed with 1% osmic acid for 3 h. The fixed polyester film was washed with buffer and dehydrated for 30 min with alcohol in gradient concentrations until completely dry. The dried sample was fixed on a sample holder, sprayed with gold, and observed and photographed under an emission scanning electron microscope.

#### Cell transmembrane resistance detection

The TEER of cells after 3, 6, 9, 12, 15, 18, and 21 d of inoculation were measured using a resistance meter. The TEER was calculated as follows:


TEER =(R assay group−R blank group)×A

where A is the cell monolayer membrane area (1.12 cm^2^), the unit is Ω·cm^2^.

#### Permeability experiment of Caco-2 cell monolayer model

The buffer was used to configure the standard solution of sodium fluorescein in gradient concentrations. The OD of sodium fluorescein was determined at 492 nm and the standard curve was made. The absorption rate of fluorescein sodium was evaluated using the monolayer model. The original culture medium in the Transwell transport chamber was discarded, cells were washed with PBS, and placed in an incubator for balance (buffer was added to each well). Then, 0.5 mL 10 μg/mL sodium fluorescein solution was added to the AP side of the chamber and 1.5 mL PBS (preheated to 37°C) was added to the BL side. The Transwell chamber was subsequently placed in an incubator. Every 30 min, a certain volume of buffer was removed from the lower chamber. The OD of sodium fluorescein was measured at 492 nm and the transmittance was calculated as follows:


$$Papp\;=\;\frac{dQ}{dt\times A\times C_{0}}$$

where dQ/dt is the transmittance of sodium fluorescein per unit time, A is the area of the Transwell chamber floor, and C_0_ is the concentration of the experimental group.

### Caco-2 cell transport experiment

The cell transport experiment was carried out using the Caco-2 cell monolayer model with transmembrane resistance and sodium fluorescein transmittance. The original medium was discarded and the cells were gently rinsed with PBS buffer (preheated to 37°C) two or three times to remove impurities on the cell surface. Then, the transmittance of sodium fluorescein was measured; the blank experiment was run at the same time. The culture plates were incubated and the liquid on both sides after each 30 min interval was collected in a centrifuge tube and diluted to a suitable concentration. The liquid was filtered with a 0.22-μm needle filter. The calcium content was detected by a calcium chromogenic detection kit. The calcium transport volume was calculated using the following formula:


$$B_{n}\;=\;0.5\;\times \;A_{n}\;+\;0.05\times \;\sum_{k=1}^{n-1}A_{k}$$

where Bn is the calcium content of 0.5 mL buffer on the BL side at different time points, μg is the calcium concentration on the BL side at different time points, and n is an independent variable (1, 2, 3, and 4 represent 30, 60, 90, and 120 min, respectively).

### Caco-2 cell pretreatment

Caco-2 cells were inoculated in cell culture plates according to the appropriate density. After the cells were full, they were treated with the cell culture medium containing phosphate at a concentration and divided into the control, positive control, and fish sausage groups with three repeats of each group. After the cells met the requirements of the follow-up experiment, the corresponding proportion of phosphate buffer was added according to the bottom area of the cell culture plate. Caco-2 cells were gently scraped off with a cell scraper on the ice box, transferred to a frozen cell tube, and placed in liquid nitrogen. After quick freezing in liquid nitrogen, Caco-2 cell samples were mailed to the Beijing Huada Protein Research and Development Center Co., Ltd. (China) for proteomic analysis.

### Caco-2 cell protein extraction

An appropriate amount of cell samples was added to the lysate and placed under ultrasonication for 5 min to facilitate cleavage {the lysate buffer included 8 m urea, 30 mM 2-[4-(2-hydroxyethyl) piperazin-1-yl] ethanesulfonic acid (HEPES), 1 mM phenylmethanesulfonyl fluoride (PMSF), 2 mM EDTA, and 10 mM DTT}. The supernatant was obtained after centrifugation at 20,000 g for 30 min. The final concentration of the solution after pyrolysis was adjusted to 10 mmol/L by adding DTT. After bathing at 56°C for 1 h, IAM was added to adjust the final concentration to 55 mmol/L and placed in a dark room for 1 h. At the end of the static placement, pre-cooled acetone was added and precipitated at −20°C for >3 h. Then, the supernatant was discarded after centrifugation at 4°C and 20,000 g for 30 min. After the addition of resoluble buffer, the final concentration of the solution was adjusted and placed under ultrasonication for 3 min. After centrifugation at 4°C and 20,000 g for 30 min, the supernatant was obtained and the extracted proteins were quantified using the Bradford method.

### Caco-2 cell protein digestion

Using an appropriate amount of cell sample, the lysate was added and the sample was placed under ultrasonication for 5 min to facilitate lysis. After centrifugation, the supernatant was obtained. Dithiothreitol and iodoacetamide were added to the supernatant to adjust the final concentration of the solution after pyrolysis. After static placement, acetone was added to the sample to precipitate at a low temperature and the supernatant was discarded after centrifugation. The final concentration was adjusted by adding resoluble buffer and ultrasonication was used to assist with solubilization. The supernatant was obtained after centrifugation and the extracted proteins were quantitatively analyzed using the Bradford method.

### Mass spectrometry detection

After balancing the TMT labeling reagent at room temperature, 41 μL acetonitrile was added then mixed for 1 min and centrifuged. A labeling reagent at room temperature was added to the redissolved peptide, mixed, and left at room temperature for 1 h. Then, 8 μL 5% hydroxylamine was added and placed at room temperature for 15 min. The sample was mixed and vacuum dried.

An appropriate amount of protein was added to the ultrafiltration tubes from each group of samples and the waste liquid was discarded after centrifugation. Tetraethyl ammonium bromide buffer was added for centrifugation and the precipitation was subsequently obtained. After repeating the above operations, trypsin was added to digest the extracted proteins and the protein digestion liquid was freeze-dried into a dry powder. Then, tetraethyl ammonium bromide (TEAB) was added to each tube to redissolve the peptides.

After the TMT labeling reagent reached room temperature, 41 μL acetonitrile was added, mixed for 1 min, and centrifuged. The labeling reagent was added to the redissolved peptide, mixed well, and placed at room temperature. After adding hydroxylamine, the sample was mixed and vacuum dried after standing at room temperature.

The purified samples were detected using a Q-Exactive mass spectrometer. The original MS data were searched in the database and the results were filtered with a false discovery rate (FDR) < 1%. The specific test parameters are shown in Tables 1–3.

**Table 1:** The parameter of mass spectrometer.

**Parameter name**	**Parameter**	**Parameter value**
Ion mode	Polarity	Parameter value
Parent ion scanning range	MS scan range	350–2,000 m/z
Secondary resolution	Resolution	17,500
Capillary temperature	Capillary temperature	320°C
Source voltage	Ion source voltage	1,800 V
Fragmentation mode	MS/MS acquisition modes	Higher collision energy dissociation (HCD)
Collision energy normalization	Normalized collision energy (NCE)	28

**Table 2:** The parameter of Dionex ultimate 3,000 nano LC system.

**Item**	**Parameter**
Nano LC trap	Acclaim pepmap 100; 150 μm × 2 cm nanoviper C18 5 μm 100A
Nano LC column	C18 5 μm 75 μm × 15 μm 300A
Solvent A	0.1% formic acid 2% ACN 98% water
Solvent B	0.1% formic acid 2% water 98% ACN
Flow rate	0.4 μL/min

**Table 3:** The parameter of identification and retrieval.

**Parameter name**	**Parameter**	**Experimental options**
Mascot version number	Mascot version	2.3.0
Fixed modification	Fixed modification	Carbamidomethyl (C), itraq 8 plex (K), itraq 8 plex (N-term)
Variable modification	Variable modification	Oxidation (M), Gln → Pyro-Glu (N-term Q), Itraq 8 plex (Y)
Primary quality deviation	Peptide tol	15 ppm
Secondary quality deviation	MS/MS tol	20 mmu
Maximum allowable missed cleavage	Max missed cleavages	1
Enzyme typ	Enzyme	Trypsin
Database	Database	2019-uni-human; Time files compressed: 2019.10.22; Number of sequences: 172097

### Biological and data analyses

Based on the identified differential protein IDs between the control and fish sausage groups, we annotated the related proteins in the GO database. The corresponding proteins were listed according to three components in the GO database and made into a statistical chart (34). Through a KEGG pathway analysis, the differential proteins were annotated and their distributions in the related pathways were directly observed (35). The differential proteins were also searched in the String database (medium confidence, 0.400) and selected to further construct a protein interaction network (36). The data were processed and analyzed using Thermo Fisher Proteome Discoverer v1.3 and Mascot v2.3.01 software.

## Results

### Activity of Caco-2 cells

High phosphate levels can damage endothelial cells. The intake of processed foods with high phosphate additives leads to higher levels of phosphorus in the human body, which increases the risk of cardiovascular disease (37). Therefore, a concentration range of normal human blood phosphorus levels was selected to evaluate the response of Caco-2 cells to increased extracellular phosphate levels.

**Figure 1:**
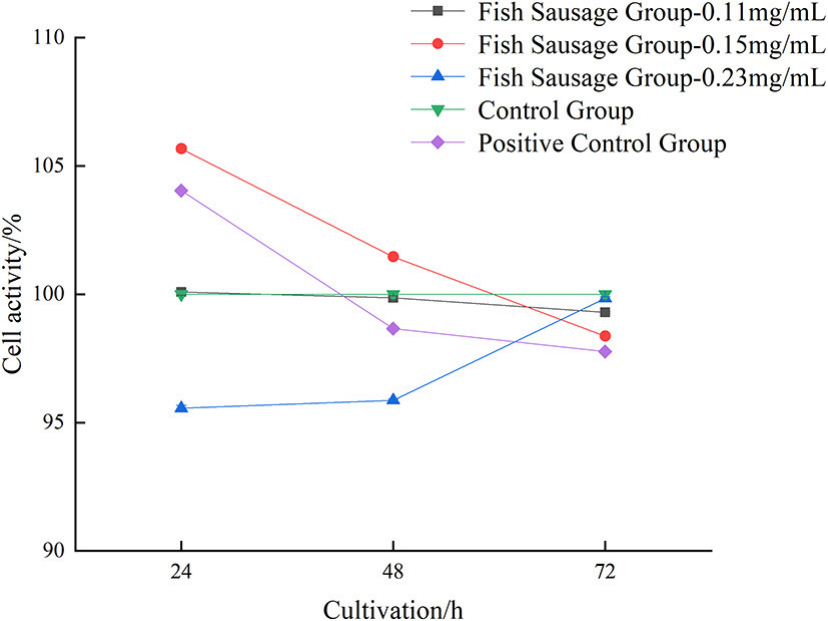
The impact of *in vitro* digestive juice of bass on the viability of Caco-2 cells.

The digestive juice of sea bass sausages under different phosphate concentrations over time greatly affected the proliferation of Caco-2 cells (Figure 1). After Caco-2 cells were treated with 0.23 mg/mL perch sausage digestive juice for 24, 48, and 72 h, the Caco-2 cell activity increased significantly over time and was the highest after 72 h (99.84%). Compared to the control group, the digestive liquid of sea bass sausages with higher or lower phosphate concentrations inhibited the proliferation of Caco-2 cells, but when the concentration of phosphate was 0.13 mg/L, the digestive liquid of 0.23 mg/mL of sea bass sausages promoted the proliferation of Caco-2 cells. Therefore, in the subsequent experiment, 0.23 mg/mL was selected for the experimental group.

### Morphological observation of Caco-2 cells

The Caco-2 cell line was first established by Fogh et al. (38). while screening the cytotoxicity of antineoplastic drugs and studying the mechanism of drug resistance. Over time, Caco-2 cells gradually and spontaneously differentiate into dense cell monolayers. The structure, morphology, and function of cells at this stage are similar to human intestinal epithelial cells. One side of the brush marginal membrane differentiate into microvilli, while the other side of the serosa differentiate into the basal surface (39, 40).

**Figure 2:**
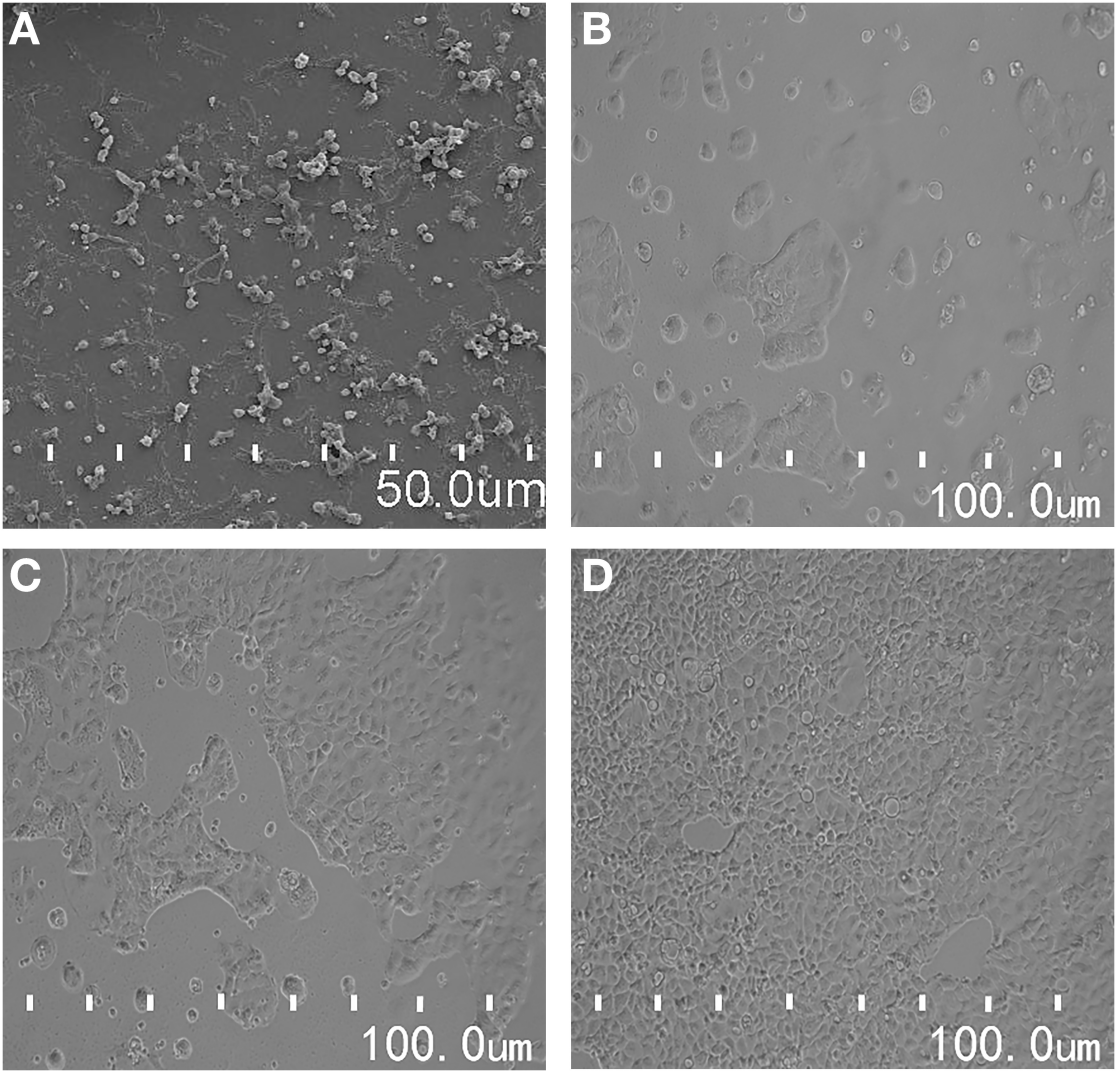
**(A)** The morphological observation of Caco-2 cells by scanning electron microscope; **(B–D)** is the morphological changes of Caco-2 cells in different culture time.

After 21 d, the morphology of Caco-2 cells was observed under an emission site scanning electron microscope. Caco-2 cells were complete and dense, and in a good state at this time (Figure 2A). The next step of the cell transmembrane resistance and sodium fluorescein transmittance test was subsequently carried out. After Caco-2 cells were inoculated on a cell plate for 24 h, the distribution of cells was sparse and some cells were still round (Figure 2B). Caco-2 cells began to fuse slowly 48 h after inoculation (Figure 2C). After 72 h, Caco-2 cells gradually formed into a state of close distribution and grew well (Figure 2D). These results indicated that the cells formed a dense cell monolayer within 21 d of culturing. In a previous study, after continuous culturing for 22 d, Xiang et al. initially established a uniform and dense, tight junction into a seamless single cell layer (41). Our findings were slightly different.

### Determination of electrical resistance of Caco-2 cell monolayer model

**Figure 3:**
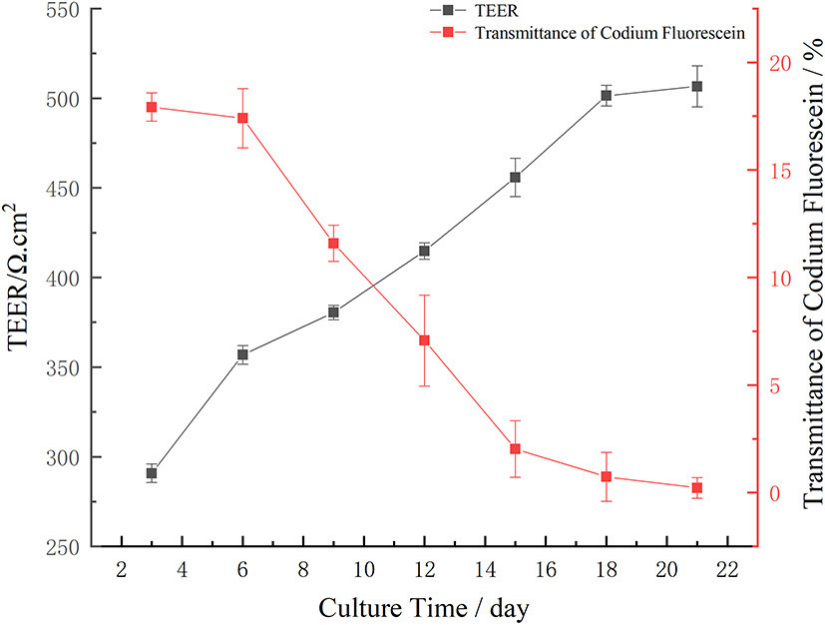
Trend of monolayer transmembrane resistance of Caco-2 cell during different culture periods, and transmittance of fluorescein sodium in different culture time: Transmittance of sodium fluorescein from AP side to BL side.

Cell transmembrane resistance refers to the ability of the passive diffusion of ionic charges in epithelial cells. Generally, transmembrane resistance should be 200–1,000 Ω cm^2^ (42). In this study, the transmembrane resistance of Caco-2 cells increased over time (Figure 3). Specifically, after 3, 6, 9, 12, 15, 18, and 21 d, the transmembrane resistance of Caco-2 cells mainly increased. From day 3 to 6, the TEER value increased rapidly from 290.83 to 356.91 Ω cm^2^. From day 6 to 18, the TEER value steadily increased from 356.91 to 501.39 Ω cm^2^. On day 12, the TEER value was >400 Ω cm^2^. After 18 d, the TEER value tended to be stable until day 21 when it was ~500 Ω cm^2^. These results are consistent with Li et al. (43). Who found that the transmembrane resistance of cells was >500 Ω cm^2^ after 21 d. These findings showed that the compactness of Caco-2 cells was good within 21 d and was thus selected for use in the next experiment.

### Verification of sodium fluorescein permeability in Caco-2 cell monolayer model

Intestinal mucosal permeability is an important characteristic of the intestinal tract that determines the selectivity of intestinal mucosa for nutrients entering the blood and lymphatic circulation and depends on epithelial cell characteristics and a tight junction complex (44). Intestinal permeability refers to the non-mediated intestinal permeation of medium-sized hydrophilic molecules without the assistance of carrier systems (45). Intestinal permeability can be evaluated by measuring the transmembrane resistance or extracellular markers, such as sodium fluorescein, mannitol, and phenol red, among others. The linear regression equation for the absorbance of sodium fluorescein is as follows:


$$y\;=\;0.0285x\;+\;0.0141\;(R^{2}\;=\;0.9998)$$

where “y” is the absorbance value and “x” is the concentration of sodium fluorescein (μg/mL).

The transmittance of sodium fluorescein from the AP side to the BL side is shown in Figure 3. The transmittance of sodium fluorescein decreased over time. From day 3 to 6, the transmittance of sodium fluorescein decreased slowly, which was 17.920 and 17.403%, respectively. The transmittance of sodium fluorescein decreased significantly from 17.403% on day 6 to 2.018% on day 15. The transmittance of sodium fluorescein decreased slowly from day 15 to 0.220% on day 21. Over time, the monolayer model of Caco-2 cells found that the sodium fluorescein permeability decreased gradually, then gradually decreased, which indicated that the monolayer Caco-2 cell model had good compactness and could be used for subsequent transport experiments.

### Calcium transport experiment of Caco-2 cells

**Figure 4:**
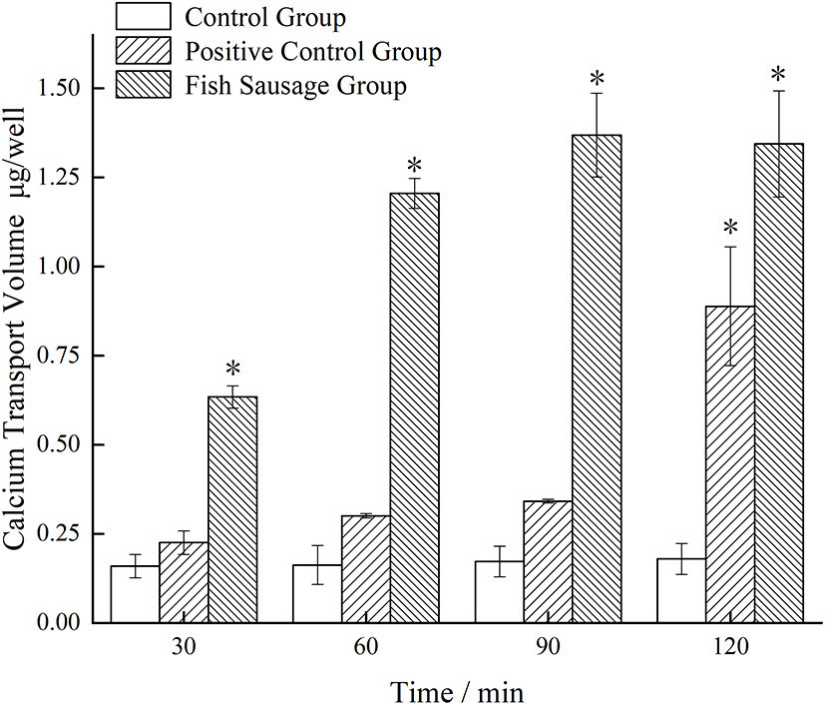
The calcium transport through Caco-2 cell monolayer model of simulated digestive fluid of bass sausage *in vitro*. Comparison among the three groups: *P < 0.05.

Figure 4 shows the changes in calcium transport in the monolayer Caco-2 cell model treated with simulated digestive juice of sea bass sausages *in vitro*. Compared to the control group, the amount of calcium transport in the fish sausage group significantly increased after transport through the monolayer Caco-2 cell model (*P* < 0.05). Over time, the calcium transport amount also increased. After incubating for 90 min, the amount of calcium transport in the fish sausage group was significantly higher than the control group (*P* < 0.05). The calcium transport amount in the fish sausage group reached its maximum value of 1.369 μg/well.

The calcium transport volume of the control and positive control groups did not change significantly over time; the calcium transport amount was relatively small, which may be because the control group only contained a complete cell culture medium. Calcium ions in a complete medium can maintain osmotic pressure balance in the cells, but their content is lower, thus, the amount of calcium transport in the control group was lower. One reason why the amount of calcium transport in the positive control group may have been higher is that the control group may have had high K^+^ concentrations that affected the cell membrane potential, thereby affecting the voltage-dependent calcium channel in the membrane. These conditions lead to an influx of extracellular calcium or release of calcium from the intracellular calcium pool, resulting in a rapid increase in the intracellular calcium content (46). However, to maintain a lower intracellular calcium content, calcium ions entering the cytoplasm can be returned to the extracellular environment or calcium pool through the Ca^2+^-ATP enzyme (47). To summarize, there is a certain content of calcium present in the digestive juice of sea bass sausages simulated *in vitro* and the amount of promoting calcium transport was higher than the control and positive control groups.

### Identification after protein extraction

Table 4 shows the protein quantification results in Caco-2 cells after treatment. The linear regression equation for protein quantification is as follows:


$$y\;=\;0.1616x\;+\;0.0077\;(R^{2}\;=\;0.9924)$$

where “y” is the absorbance value and “x” is the protein concentration (μg/μL).

**Table 4:** The quantitative results of protein samples.

**Sample name**	**Positive control group 1–1**	**Positive control group 1–2**	**Fish sausage group 1–1**	**Fish sausage group 1–2**	**Control group 1–1**	**Control group 1–2**
Concentration (μg/μL)	1.18	1.00	1.09	0.99	1.69	1.35
Sample volume (μL)	200	200	200	200	200	200
Total protein (μg)	236	200	218	197	338	270

According to the quantitative results, the total amount of extracted protein was about 200 μg. it can meet the amount needed for protein enzymatic hydrolysis. Thus, the MS detection of differential proteins could be carried out.

### Protein qualitative analysis

#### Display of qualitative results

For the TMT quantitative detection of proteins, there were six groups of samples, the control, positive control, and fish sausage group with two biological repeats of each group. 179,396 spectrums were generated after MS detection. According to the identification results, the number of matching peptides identified by the data was 25,125, the number of matching spectra was 63,046, and the number of protein groups was 4,959 (Table 5).

**Table 5:** The information of qualitative result.

**Item**	**Number of tests**
Sample information	6
Repeat experiment	1
Matching spectrum	63,046
Matching number of peptides	25,125
Number of proteomes	4,959

#### Analysis of qualitative results of extracted protein

**Figure 5:**
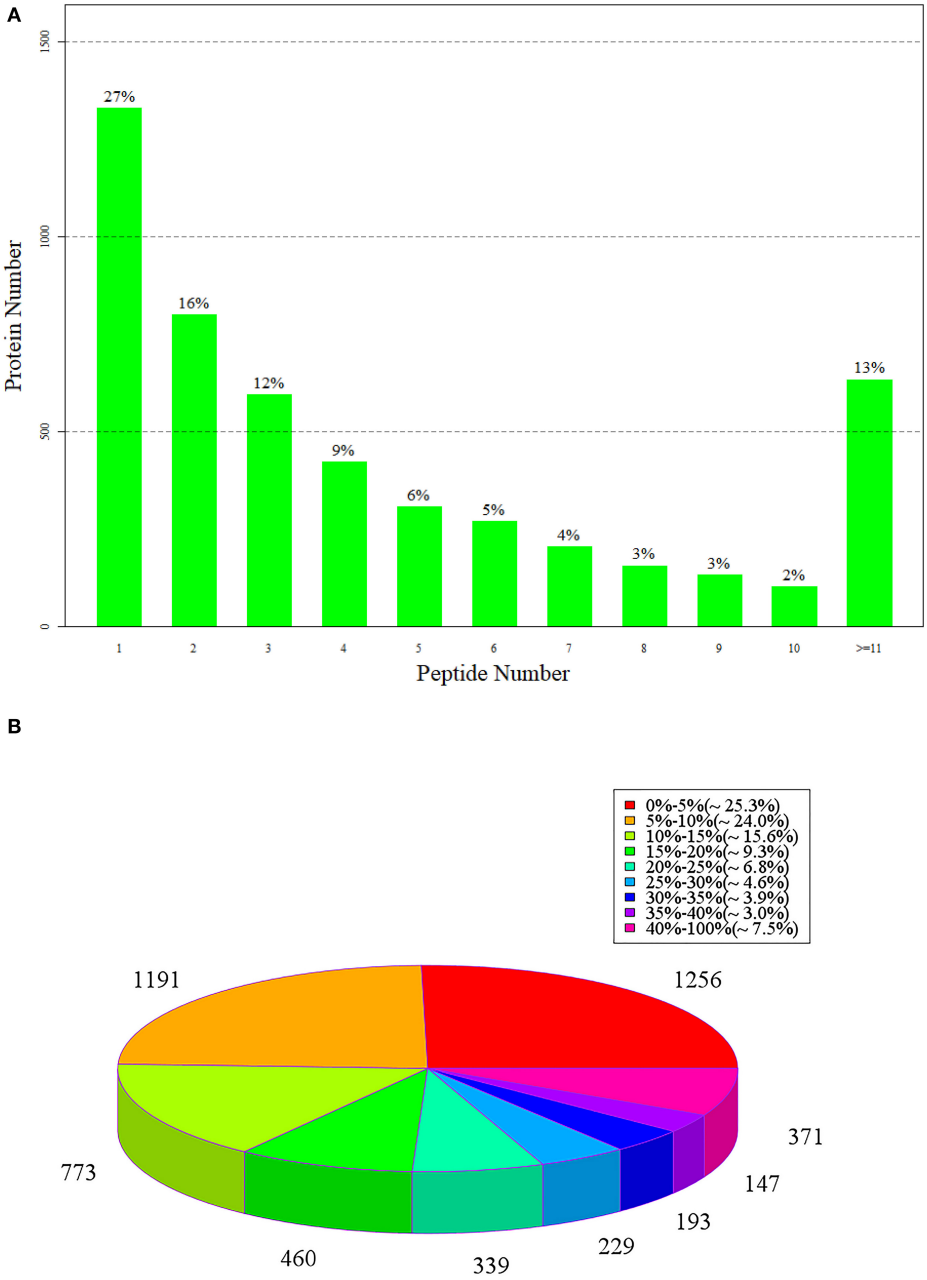
**(A)** The number of peptides matched to proteins; **(B)** is the statistical diagram of protein identification coverage.

The number of protein-matching peptides can be used to analyze the specific number of peptides contained in the identified proteins. The greater the number of matching peptides, the lower the number of proteins corresponding to the number of peptides. In the 4,959 groups of identified proteins, 27% of the proteins contained one peptide and the number of proteins composed of two and three peptides accounted for 16 and 12%, respectively (Figure 5A).

Figure 5B shows the statistics of the corresponding coverage of the identified proteins, in which, different colors represent the coverage of different proteins and the number above each color represents the specific number of proteins. Protein coverage refers to the proportion of the number of amino acids identified in a protein to the original number of amino acids in the protein (48). In 4,954 groups of identified proteins, there were 1,256 groups of proteins with a corresponding coverage rate of 0–15%, accounting for 25.3% of the total proteins, and 1,191 groups with a coverage rate of 5–10%, accounting for 24%. There were 733 groups of proteins with a coverage rate of 10–15%, which accounted for 15.6%. The coverage of most proteins was small and mainly concentrated at 0–13%.

### Protein quantitative analysis

**Figure 6:**
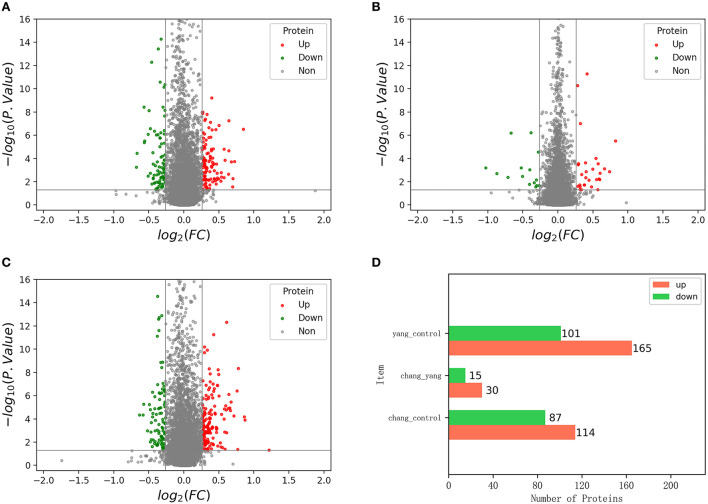
The volcano map of differentially expressed proteins; **(A)** is the volcano map of control group vs. fish sausage group; **(B)** is the volcano map of positive control group vs. fish sausage group; **(C)** is the volcano map of control group vs. positive control group; **(D)** is the number of up-and down-regulated differential proteins in each comparison group.

The volcano map shows the distribution of the identified differential proteins according to their multiples of change and *P*-values. Figures 6A–C show the volcano map of differential proteins among groups, in which, red dots represent the expression of significant differential proteins and gray dots represent the expression of non-significant differential proteins.

An ANOVA was conducted, using *P* < 0.05 and difference multiple >1.2-times as the screening criteria to determine significant differences between the screened differential proteins (Figure 6D). The number of up-regulated and down-regulated differential proteins in each comparison group was calculated. Red represents the number of up-regulated proteins and green represents the number of downregulated proteins. Compared with the control group, the total number of differential proteins detected in the fish sausage group was 201, including 114 up-regulated proteins and 87 down-regulated proteins. A total of 45 differential proteins were detected in the fish sausage group compared with the positive control group, including 30 up-regulated proteins and 15 down-regulated proteins. Compared with the positive control group, the total number of differential proteins detected in the control group was 266, including 165 up-regulated proteins and 101 down-regulated proteins. Results revealed that both the positive control and fish sausage group significantly affected the protein expression levels in Caco-2 cells.

### GO analysis results

#### Go notes

**Figure 7:**
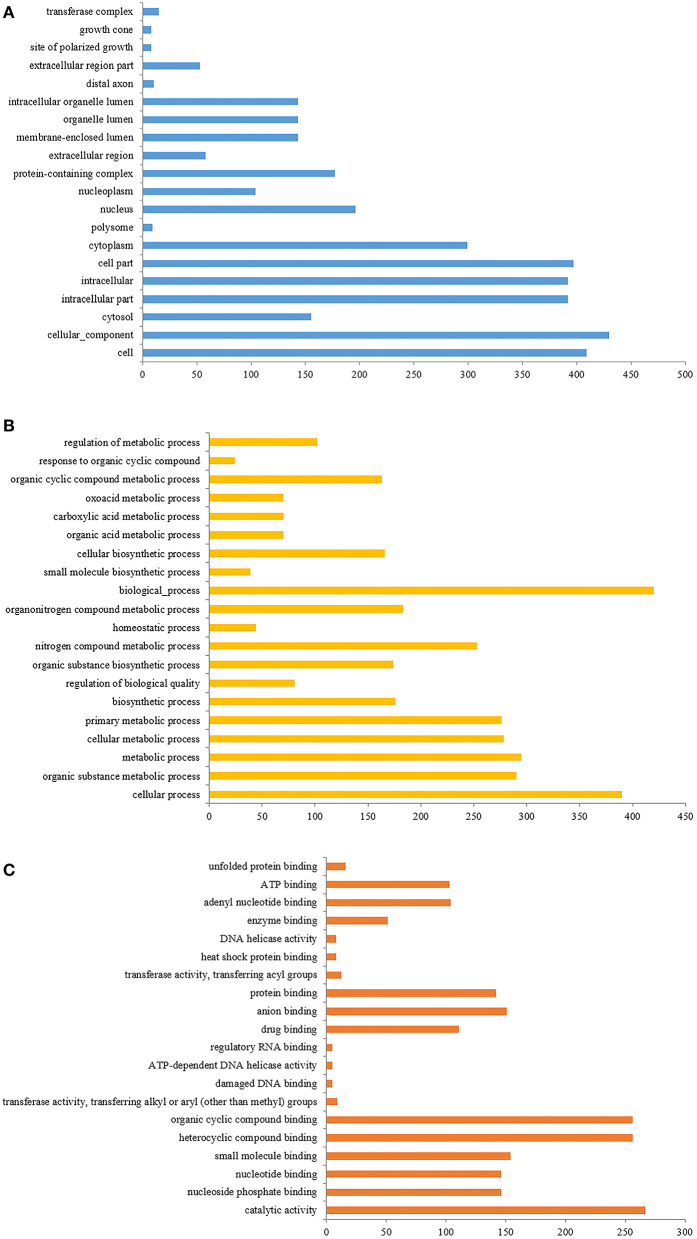
**(A)** The GO enrichment analysis of differentially expressed proteins (cell components); **(B)** is the GO enrichment analysis of differentially expressed proteins (biological process); **(C)** is the GO enrichment analysis of differentially expressed proteins (molecular function).

The identified differential proteins were compared to the Uniprot database using David software for classification and annotation. The identified protein GO annotation classifications are mainly divided into three parts: cellular components (CC), molecular functions (MF), and biological processes (BP) (Figures 7A–C) (49). In BP, the functions with more annotations were mainly concentrated in biochemical processes, cell metabolic processes, metabolic processes, and nitrogen compound metabolic processes, while in CC, the annotated functions mainly included cellular components, intracellular components, and cytoplasm. In MF, the annotated functions mainly focused on catalytic activity, heterocyclic compound binding, and organic ring compound binding. Based on the GO annotation analysis of the top 50 differential proteins, in order of significance, 21 components were concentrated in BP, 18 were concentrated in CC, and the remaining 11 were concentrated in MF.

#### GO enrichment analysis

**Figure 8:**
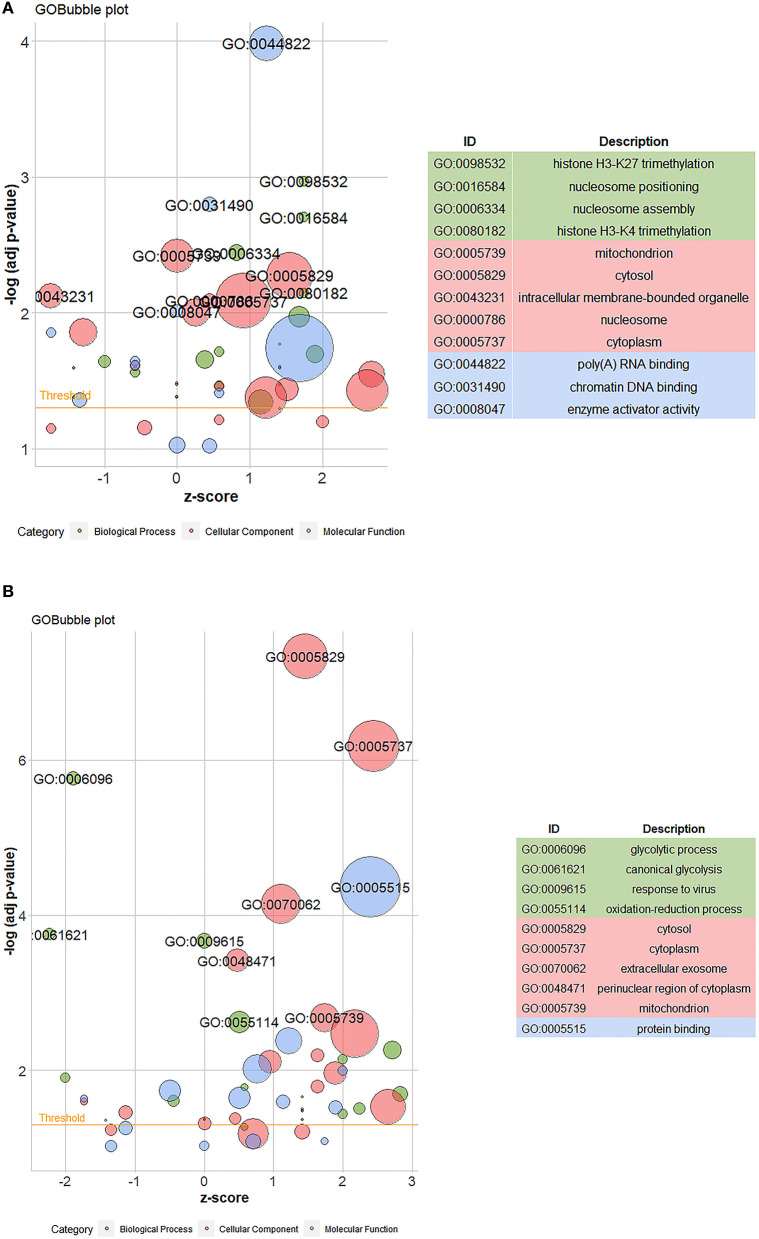
**(A)** The GO bubble map of differential proteins between control (0 mg/mL phosphate residue) and fish sausage group (0.13 mg/L phosphate residue); **(B)** is the GO bubble map of differential proteins between positive control (1 mg/L phosphate residue) and control group (0 mg/L phosphate residue).

*P*-value < 0.01 is the selection ranges of differential proteins. The GO enrichment analysis of the differential proteins identified in Caco-2 cells treated with compound phosphate was carried out using David software. Figures 8A,B show the GO analysis bubble diagrams of the differential proteins identified between the control and fish sausage groups, and the control and positive control groups. Z-score represents the overall expression trend of differential proteins enriched to this function, with Z-score >0 indicating up-regulation and Z-score < 0 indicating down-regulation. Red represents the CC component, blue represents the MF component, and green represents the BP component. The circle size represents the number of differential proteins enriched in a given function; the larger the circle, the greater the number of differential proteins.

The top 10 differential proteins were mainly enriched in CC and the main enriched GO items included mitochondria, cytoplasm, organelles, and nucleosomes on the inner surface of the cell (Figure 8A). The second was enriched in BP and mainly included histone H3–K27 trimethylation, nucleosome localization, nucleosome assembly, and histone H3–K4 trimethylation. The differential proteins between the control and positive control groups were mainly enriched in CC, including cytoplasm, mitochondria, cytoplasm, exosomes, and perinuclear regions of the cytoplasm, and in BP, including glycolysis, typical glycolysis, virus reaction, and redox process (Figure 8B). The functions of the differential proteins between the control and fish sausage groups and the control and positive control groups were mostly enriched in areas with a Z-score >0, indicating that the overall expression of differential proteins was upregulated (Figures 8A,B). Thus, compound phosphate can promote this series of functions, these include: poly (A) RNA binding, cytosol, nucleosome, cytoplasm, histone H3–K27 trimethylation, nucleosome positioning, nucleosome assembly, histone H3–K4 trimethylation, protein binding, perinuclear region of cytoplasm, mitochondrion, extracellular exosome and oxidation-reduction process.

### KEGG pathway analysis results

**Figure 9:**
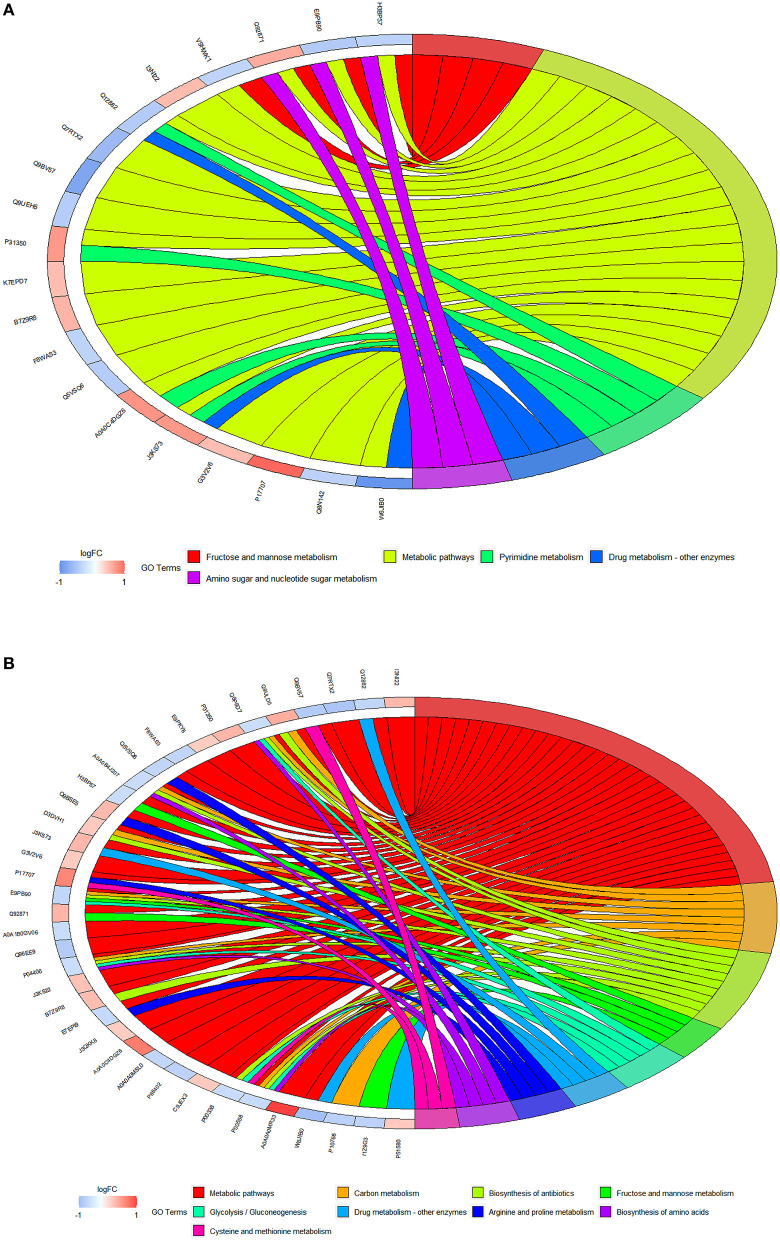
**(A)** The GO chord diagrams of differential proteins between control (0 mg/L phosphate residue) and fish sausage group (0.13 mg/L phosphate residue); **(B)** is the GO chord diagrams of differential proteins between control (0 mg/L phosphate residue) and positive control group (1 mg/1L phosphate residue).

The KEGG pathway enrichment analysis of differential proteins was carried out using David software. Figures 9A,B show the pathway chord diagrams of the differential proteins between the control and fish sausage groups, and the control and positive control groups. The differential proteins between the control and fish sausage groups were mainly enriched in the metabolic pathway, followed by fructose and mannose metabolism and pyrimidine metabolism. The differential proteins between the control and positive control groups were mainly enriched in metabolic pathways, antibiotic biosynthesis, and carbon metabolism. The above data suggest that complex phosphate may greatly affect the transport of calcium ions through the metabolic pathway, then the radiation of the biosynthetic signaling pathway may thereby affect the transport of calcium ions.

Table 6 shows the differential proteins identified in the three groups. Through biological process and pathway analysis, it was found that the key differential proteins included Q5EBL8, P02795, Q9GZT9, Q96PU5, and Q9P0W0, among them, Q5EBL8 and P02795 are closely related to metal ion transport, Q9GZT9, Q96PU5, and Q9P0W0 were enriched, and they were different from the positive group. P02795 (Metallothionein-2, MT-2) is an important member of the metallothionein family. Its main physiological characteristics include metal ion binding and reducibility, and it can reversibly bind to divalent ions, such as zinc, copper, iron, and cadmium, to maintain metal ion balance *in vivo* (50). The concentration of calcium affected the expression of MT-2. In keratinocyte serum-free medium (K-SFM), 4 mM Ca^2+^ significantly decreased the expression of MT-1/2 proteins in RWPE-1 cells exposed to Cd^2+^ (51). The expression of MT-2 in the positive control and fish sausage groups was down-regulated, which may be due to the decrease in MT-2 expression due to the increased calcium concentration. In the KEGG analysis, P02795 was enriched. The enriched pathway was mineral absorption; phosphate may affect the absorption of calcium through the mineral absorption pathway. Q5EBL8 (PDZ Domain-Containing Protein 11, PDZD11) is a PDZ protein that interacts with plasma membrane calcium ATPase (52). PDZD11 interacts with Ca^2+^-ATPase (PMCA) and affects the biology of PMCA (53). PDZD11 was down-regulated in the positive control and fish sausage groups, indicating that phosphate and fish sausage digestive fluid containing phosphate affected the expression of PDZD11 (Table 6). Q9P0W0 (Interferon-Kappa, IFNK) is a type I interferon with 30% similarity to other type I interferon subclasses (54). In addition to antiviral activity, interferon is involved in a wide range of cellular functions, such as inhibiting the proliferation of normal and tumor cells, stimulating natural killer cells, increasing the expression of major histocompatibility complex antigens, and stimulating tumor antigens (55). The fish sausage and positive control groups promoted IFNK expression, and the positive control group upregulated the expression of IFNK. Therefore, phosphate promoted IFNK expression. Q96PU5 (E3 Ubiquitin-Protein Ligase NEDD4-like, NED4L) is a highly conserved Het E3 ligase (56). NEDD4L mediates the ubiquitination of epithelial Na/K/2Cl cotransporter NKCCI/SLCI2A2 and inhibits the cell surface expression of NKCCI/SLCI2A2 (57). NEDD4L inhibits tumor growth through the proliferation of ubiquitinated proteins and plays an important role in the occurrence and development of colorectal cancer (58). In this study, phosphate promoted the expression of NED4L, and fish sausages containing phosphate significantly increased NED4L expression. Q9GZT9 (Eglninehomolog1, EGLN1, also known as PHD2) is an oxygen-sensitive factor that regulates the expression of downstream genes by sensing the oxygen content in cells, thereby affecting important physiological processes, such as glucose uptake, metabolic regulation, angiogenesis, and cell cycle (59). Previous studies have shown that EGLN1 expression is closely related to the occurrence and prognosis of colorectal cancer (60). In the Caco-2 cell model, we found that the fish sausage and positive control groups inhibited EGLN1 expression.

**Table 6:** The comparison of the main differential proteins among the three groups.

**Accession**	**Description**	***P*-value**	**Positive vs. control**	***P*-value**	**Fish sausage vs. control**	***P*-value**	**Fish sausage vs, positive**
Q5EBL8	PDZ domain-containing protein 11	0.00493679	0.8165	0.0000625	0.809	0.36	1.045
Q96PU5	E3 ubiquitin-protein ligase NEDD4-like	0.001600833	1.299	0.027334657	1.2535	0.97	1.0033
Q9P0W0	Interferon kappa	1.49E-08	1.412	0.000000318	1.373	0.54	0.9795
Q9GZT9	Egl nine homolog	0.0000153	0.717	0.00000302	0.6775	0.32	0.956
P02795	Metallothionein-2	3.77E-09	0.802	0.000000427	0.7485	0.01	1.089

In the KEGG analysis, Q9GZT9, Q96PU5, and Q9P0W0 were differential proteins identified in the positive control and fish sausage groups, which were enriched in the fish sausage group, but not in the positive control group. The enrichment pathways included hypoxia-inducible factor-1 (HIF-1) signaling pathway, ubiquitin-mediated protein hydrolysis, endocytosis, and Janus kinase-signal transducer, an activator of the transcription (JAK-STAT) signaling pathway. The HIF-1 signaling pathway plays an important role in autoimmune diseases (51–63). Endocytosis and ubiquitin-mediated proteolysis belong to the cellular immune pathway (64). The JAK-STAT signaling pathway is related to cell development, cell growth, and survival, thus playing an important role in immune functions. For example, STAT proteins play an important role in the differentiation of T helper lymphocytes (65). Therefore, the fish sausage group may have affected the immune-related function of the cells.

## Conclusions

In this study, a monolayer model of Caco-2 cells cultured *in vitro* was established to explore the transport of calcium in the digestive juice of sea bass. Results revealed that the sausage digestive fluid of sea bass at different phosphate concentrations and culture times greatly affected the proliferation of Caco-2 cells. Cell culture medium containing 0.23 mg/mL sea bass sausage digestive solution effectively increased the proliferation activity of Caco-2 cells. After 21 d of culture, Caco-2 cells formed a dense monolayer structure and the monolayer model had good permeability. According to the calcium transport data, after Caco-2 cells were treated with perch sausage juice, the amount of calcium transport in the digestive juice increased significantly over time. After 90 min, calcium transport reached its maximum, indicating that compound phosphate effectively promoted intracellular calcium transport.

The TMT protein quantitative technique was used to detect the differential proteins of Caco-2 cells treated with compound phosphate. Results revealed that the number of matching peptides identified by the data was 25,125, the number of matching maps was 63,046, and the number of protein groups was 4,959. Compared to the control group, the total number of differential proteins detected in the fish sausage group was 201, including 114 upregulated and 87 downregulated proteins. The identified differential proteins were analyzed by bioinformatics analysis. Results revealed that the differential proteins between the control and fish sausage groups were mainly enriched in CC and the overall expression of the corresponding functions enriched by the differential proteins between the control and fish sausage groups, the control, and positive control groups tended to be upregulated. Therefore, it can be inferred that compound phosphate may promote this series of functions. The differential proteins between the control and fish sausage groups were enriched in the metabolic pathway, while the differential proteins between the control and positive control groups were enriched in metabolic pathways, antibiotic biosynthesis, and carbon metabolism. Complex phosphate may affect the absorption of calcium through the mineral absorption pathway. The immune-related function of the cells in the fish sausage group may have also been affected.

## Data availability statement

The original contributions presented in the study are included in the article/Supplementary material, further inquiries can be directed to the corresponding author/s.

## Author contributions

WW and SY provided experimental design. SY provided project administration and funding acquisition. ZJ and PL performed the initial literature research. ZW and RM carried out experimental research, analyzed the data, and wrote the manuscript. XL, ZZ, and JL reviewed manuscript. All authors contributed to the article and approved the submitted version.

## Funding

We were grateful for the financial support from the National Key R&D Program of China (2018YFD0901004).

## Conflict of interest

The authors declare that the research was conducted in the absence of any commercial or financial relationships that could be construed as a potential conflict of interest.

## Publisher's note

All claims expressed in this article are solely those of the authors and do not necessarily represent those of their affiliated organizations, or those of the publisher, the editors and the reviewers. Any product that may be evaluated in this article, or claim that may be made by its manufacturer, is not guaranteed or endorsed by the publisher.
