# Supplementary figures and images for: Investigating on the influence mechanism of sausage of sea bass on calcium absorption and transport based on Caco-2 cell monolayer model
Source: Front Nutr. 2022 Oct 18;9:1046945. doi: 10.3389/fnut.2022.1046945 (PMC9623112; doi:10.3389/fnut.2022.1046945)

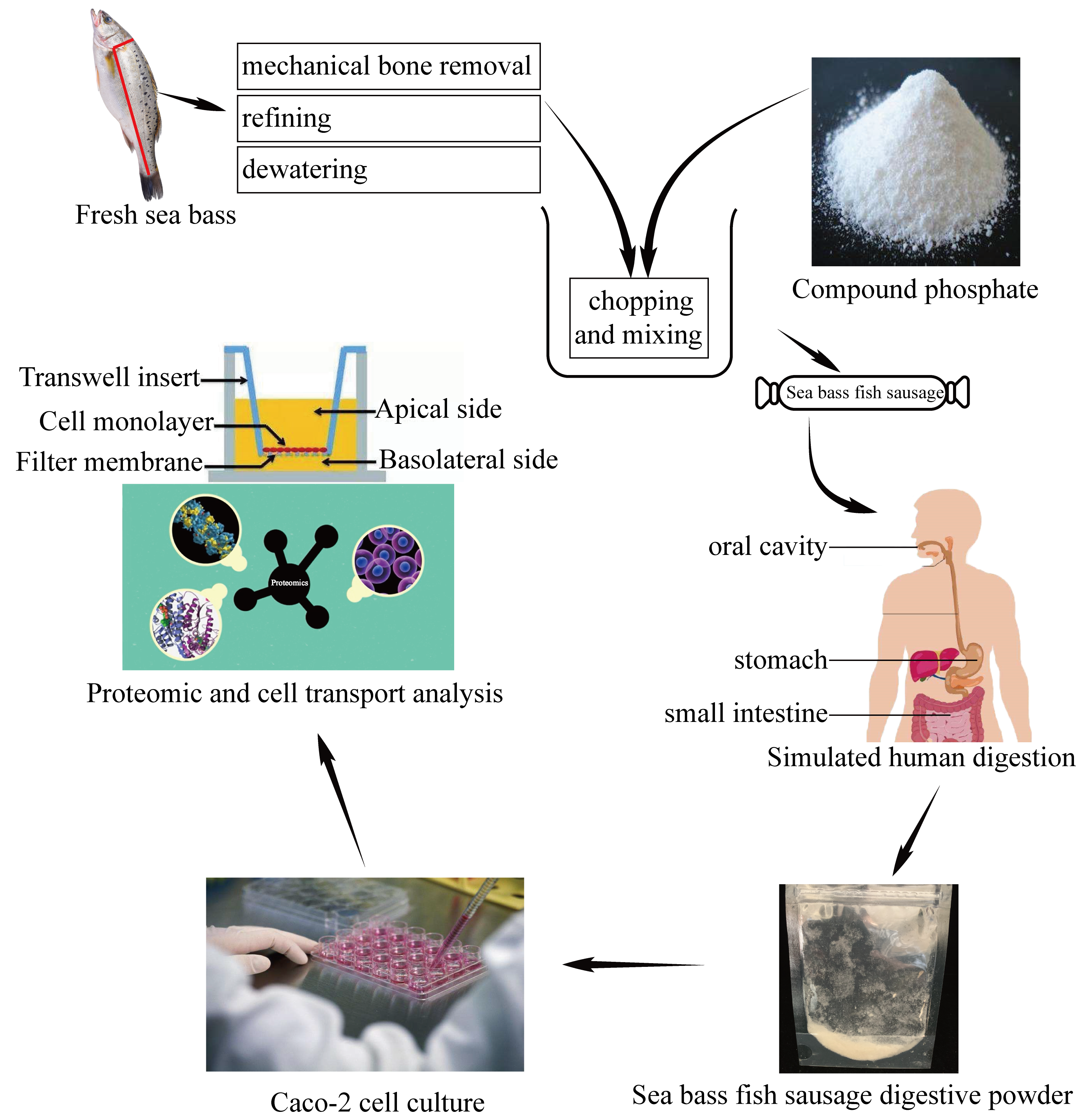

Supplement: Supplementary file 1 [file Image_1.TIF]
